# 

**Published:** 1886-09-20

**Authors:** 


### EDITORIALS AND MISCELLANEOUS.

### EDITORIAL NOTICES.

**Something Useful.**—To any one who will send us four cash subscribers, we will send as a premium one of Barry's splendid Chemical Thermometers.

R. C. WORD, *Managing Editor.*

**The Southern Medical College**, Atlanta, Ga.—This is one of the best Institutions for medical instruction in the United States. The Chairs are well and ably filled. The curriculum is complete. The building is commodious and well arranged. The dissecting room is admirably fitted up for the purpose. The College Hospital is in good running order, and every facility exists for imparting a thorough medical education. Expenses of board, tuition, etc., very moderate. For futher information address, W. P. NICOLSON, M. D., Atlanta, Ga.

DR. DEWELL GANN called at our editorial sanctum on the 10th instant. He was looking well, and, though a young member of the profession, is doing well in the practice at Smyrna, Ga.

THE MEDICAL COLLEGES OF ATLANTA.—The opening lecture of the Southern Medical College for the approaching term will be delivered at the College auditorium on Tuesday, October 5, by Prof. G. G. Roy.

DR. W. J. BURT, Secretary of the Texas Medical Association, died on the 9th of July last. He formerly resided in Georgia, whence he removed, many years ago, to Austin, Tex.

WE had the pleasure, recently, of a call from Dr. J. T. Cleveland, of Cuthbert, Ga. It is always pleasant to meet the members of the profession, and especially our subscribers. Dr. Cleveland is an intelligent and jovial member of the brotherhood.

THE STONE MOUNTAIN MEDICAL ASSOCIATION meets quarterly at Stone Mountain, Ga. Its next meeting will be on Thursday, October 7. The Physicians of DeKalb, Gwinnet, Rockdale, Newton and the counties adjacent are expected to attend.

HIGHLY IMPORTANT DISCOVERY.—The London Lancet states that Mr. Cresswell Henett has discovered a process by which Quinine can be made by synthesis at a cost of 3 pence an ounce. It was suggested in 1869 by the late Dr. Mattheson, of St. Bartholomew Hospital, and Dr. Parker, of Netley, afterwards rendered aid by his advice, so that the drug can now be manufactured from an article found in any part of the world at little cost by Mr. Henett's process.

BEAUTIFUL SATCHEL CASES FOR PHYSICIANS, SURGICAL INSTRUMENTS, ETC.—We are in receipt of a neat, compact, and beautiful black morocco satchel case from the house of E. A. Yarnall, of Philadelphia. He keeps a large and varied stock of buggy and hand cases, elastic stockings, knee-caps, anklets, abdominal supporters, splints, etc. The case sent us is a model of its class. It is a 10 x 5-inch frame, and contains eighteen 1-ounce vials, eleven 6-drachm vials, eleven 4-drachm vials—in all 40 vials—with loops for instruments, box for sundries, and a neat plush-lined case for hypodermic syringe. He keeps a variety as to size and quality, so that any one can be suited, with prices varying from $13 down to $3.25. A neat case is a great convenience and comfort to the physician. It makes a favorable impression upon his patrons, as evincing a taste and care in preparing for his profession, and thus serving as a good advertisement for himself wherever he goes.

1886.—Drs. A. F. Pharr, R. A. McQueen, I. E. Martin, G. W. Earle, to August; E. K. Bozeman, M. D. Miles, to April; Dewell Gann, to October; I. H. Claywell, T. W. Lowry, to December.

1885.—Drs. I. L. Dement, D. S. Aswell, to July; Robert Trippe, James Lofton, Elias Smyth, Abel Johnson, James A. Meyers, William Miller, Johnathan Jones, T. T. Marvin.

